# Fatty acids metabolism affects the therapeutic effect of anti-PD-1/PD-L1 in tumor immune microenvironment in clear cell renal cell carcinoma

**DOI:** 10.1186/s12967-023-04161-z

**Published:** 2023-05-23

**Authors:** Hansen Lin, Liangmin Fu, Pengju Li, Jiangquan Zhu, Quanhui Xu, Yinghan Wang, Mukhtar Adan Mumin, Xinwei Zhou, Yuhang Chen, Guannan Shu, Gaosheng Yao, Minyu Chen, Jun Lu, Lizhen Zhang, YuJun Liu, Yiqi Zhao, Jiahao Bao, Wei Chen, Junhang Luo, Xiaofei Li, Zhenhua Chen, Jiazheng Cao

**Affiliations:** 1 Department of Urology, The First Affiliated Hospital of Sun Yat-Sen University, Guangzhou, People’s Republic of China; 2 Institute of Precision Medicine, The First Affiliated Hospital of Sun Yat-Sen University, Guangzhou, People’s Republic of China; 3 Zhongshan School of Medicine, Sun Yat-Sen University, Guangzhou, China; 4 Guangdong Provincial Key Laboratory of Stomatology, Guanghua School of Stomatology, Hospital of Stomatology, Sun Yat-Sen University, Guangzhou, People’s Republic of China; 5 Department of Urology, Jiangmen Central Hospital, Haibang Street 23, Pengjiang District, Jiangmen, 529030 Guangdong China

**Keywords:** Fatty acids metabolism, Clear cell renal cell carcinoma (ccRCC), Tumor immune microenvironment, Anti-PD-1/PD-L1 therapy

## Abstract

**Background:**

Clear cell renal cell carcinoma (ccRCC) is a highly invasive and metastatic subtype of kidney malignancy and is correlated with metabolic reprogramming for adaptation to the tumor microenvironment comprising infiltrated immune cells and immunomodulatory molecules. The role of immune cells in the tumor microenvironment (TME) and their association with abnormal fatty acids metabolism in ccRCC remains poorly understood.

**Method:**

RNA-seq and clinical data of KIRC from The Cancer Genome Atlas (TCGA) and E-MTAB-1980 from the ArrayExpress dataset. The Nivolumab group and Everolimus group of the CheckMate 025 study, the Atezolizumab arm of IMmotion150 and the Atezolizumab plus Bevacizumab group of IMmotion151 cohort were obtained for subsequent analysis. After differential expression genes identification, the signature was constructed through univariate Cox proportional hazard regression and simultaneously the least absolute shrinkage and selection operator (Lasso) analysis and the predictive performance of our signature was assessed by using receiver operating characteristic (ROC), Kaplan–Meier (KM) survival analysis, nomogram, drug sensitivity analysis, immunotherapeutic effect analysis and enrichment analysis. Immunohistochemistry (IHC), qPCR and western blot were performed to measure related mRNA or protein expression. Biological features were evaluated by wound healing, cell migration and invasion assays and colony formation test and analyzed using coculture assay and flow cytometry.

**Results:**

Twenty fatty acids metabolism-related mRNA signatures were constructed in TCGA and possessed a strong predictive performance demonstrated through time-dependent ROC and KM survival analysis. Notably, the high-risk group exhibited an impaired response to anti-PD-1/PD-L1 (Programmed death-1 receptor/Programmed death-1 receptor-ligand) therapy compared to the low-risk group. The overall levels of the immune score were higher in the high-risk group. Additionally, drug sensitivity analysis observed that the model could effectively predict efficacy and sensitivity to chemotherapy. Enrichment analysis revealed that the IL6-JAK-STAT3 signaling pathway was a major pathway. IL4I1 could promote ccRCC cells’ malignant features through JAK1/STAT3 signaling pathway and M2-like macrophage polarization.

**Conclusion:**

The study elucidates that targeting fatty acids metabolism can affect the therapeutic effect of PD-1/PD-L1 in TME and related signal pathways. The model can effectively predict the response to several treatment options, underscoring its potential clinical utility.

**Supplementary Information:**

The online version contains supplementary material available at 10.1186/s12967-023-04161-z.

## Introduction

Renal cell carcinoma (RCC) is the most common subtype of primary kidney cancer and results in numerous cases and deaths worldwide [1]. Immunocheckpoint therapies (ICTs) are the most rapidly growing clinical strategy for treating RCC and provide durable clinical benefits for patients with advanced ccRCC [2]. Simultaneously, two potential immune targets PD-1/PD-L1 as prognostic markers present in ccRCC. Antibodies against PD-1, including nivolumab and pembrolizumab, serve as a useful treatment of metastatic RCC [3, 4]. Nonetheless, unlike other immunotherapy-responsive solid tumors, many RCC patients show primary or adaptive resistance and adverse events to ICTs and not all patients show complete responses [5], suggesting a further understanding of PD-1/PD-L1 mediated immunosuppression in RCC is needed to enhance treatment efficacy.

Tumor growth depends on oncogene-driven reprogramming of cell metabolism, which enables cancer cells to absorb nutrients, build macromolecules, and proliferate [6]. Increasing evidence shows that highly proliferative cancer cells have been found to increase the number of enzymes that are involved in lipid and cholesterol biosynthesis [7]. Lipid droplets stored by excess lipids and cholesterol and their quantity is related to tumor invasiveness [8, 9]. The increase of fatty acids in TME could cause the accumulation of lipid droplets and reprogramming lipid metabolism could impact indirectly on the function of immune cells and enhance tumor immunotherapy [10]. ccRCC is prominently featured with the accumulation of robust lipid as well as glycogen and associated with metabolic reprogramming for adaptation to the TME [11, 12], and different enzymes in fatty acids metabolism are potential biomarkers for diagnosis and promise for clinical effect in patients with ccRCC [13]. Due to the existence of a highly dynamic TME, and due to the glucose and fatty acids metabolism in ccRCC, this cancer could be accompanied by diverse types of resistance to immunocheckpoint therapies.

Tumor-associated macrophages (TAMs) constitute a population of immune cells with heterogeneity in TME contributing to cancer initiation and malignant progression [14]. TAMs can polarize into two phenotypes for microenvironmental signals and further modulate the TME [15]. Anti-inflammatory M1 macrophages [induced by interleukin (IL)-1β, IL-6, IL-12, IL-23 and Tumor Necrosis Factor (TNF)-α cytokines] accelerate the activation of adaptive immune responses to inhibit tumor growth, while pro-inflammatory and cytotoxic M2 macrophages [induced by IL-10, Transforming growth factor (TGF)-β, chemokine ligand (CCL)1, CCL2, CCL17, CCL18, and CCL22] exert immunosuppressive activities to promote protumorigenic activities [16, 17]. Chemokines have been described as being crucial in immune and inflammatory reactions [18], tumor-derived CCL2 expression positively correlates with TAM. C-C motif chemokine ligand 2 (CCL2) is considered as an inflammatory chemokine and mediates monocyte and macrophages migration to inflamed tissues by binding C-C chemokine receptor type 2 (CCR2) [19]. CCL2-CCR2 axis recruits tumor associated macrophages to induce immune evasion and leads to M2 macrophages polarization through PD-1 signaling pathway [20]. Correspondingly, the JAK/STAT signaling pathway is essential in M1 and M2 macrophage polarization [21], with upregulated fatty acids oxidation enhancing phosphorylation of JAK1 as well as STAT6 activation to regulate the generation of M2 macrophages [22]. In addition, arachidonic acids alter lipid raft structures to inhibit JAK1 and STAT3 phosphorylation in the ovarian cancer microenvironment where high CD206 expression is in TAMs [23]. The intricate relationship between the abnormal fatty acids metabolism and TAMs in the immune microenvironment of ccRCC has not yet been well illustrated. Therefore, the exploration of underlying relationships is crucial for future success in designing combination treatments to improve ccRCC patients’ resistance to ICTs.

In the current study, we established novel fatty-acids-metabolism-related mRNA signatures based on the TCGA cohort to contribute to the prediction about ccRCC patients’ survival prognosis. Additionally, the role of the target genes of these mRNAs in related immunotherapy of ccRCC was also clarified. The bioinformatics insights from this study will be valuable for future therapeutic development in ccRCC.

## Materials and methods

### Data acquisition

TCGA-KIRC datasets and clinical data of ccRCC (n = 526) and normal (n = 72) samples were retrieved from TGCA and were processed for subsequent analyses. The E-MTAB-1980 cohort was available on the ArrayExpress website (https://www.ebi.ac.uk/arrayexpress/). Normalized transcriptome and clinical matrix files of ccRCC patients treated with Everolimus and Nivolumab respectively in the CheckMate-025 (CM-025) cohort were collected from published articles [24]. Other normalized gene expression profiles and clinic datasets of ccRCC patients treated with Atezolizumab from a randomized phase II trial (IMmotion150 [25]) and the Atezolizumab plus Bevacizumab group of a randomized phase III trial (IMmotion151 [26]) were selected for analysis.

309 genes of fatty acids metabolism were acquired and collected from fatty acids metabolic pathways in KEGG, fatty acids metabolic genes in Hallmark and the specific genes associated fatty acids metabolism [27]. These fatty acids metabolism-related genes are provided in Additional file 2: Table S1.

### Differential expression genes identification

309 genes expression between ccRCC and normal samples in TCGA-KIRC cohort was assessed with the “limma” R package. To further visualize differentially expressed fatty acids metabolism-related genes, the heatmap as well as volcano map were drawn. The thresholds of differentially expressed gene (DEG) were as follows: the fold change (FC) of differential expression of mRNAs was |log2 fold change| ≥ 1 and False Discovery Rate (FDR) < 0.05 [28].

### Signature construction

Totally 526 ccRCC patients in TCGA-KIRC dataset included in the analysis were randomly divided into the train set (n = 395) and the validation set (n = 131) by 3:1 ratio. Based upon the train set, the fatty acids metabolism-related DEGs were further analyzed through univariate Cox proportional hazard regression and simultaneously the least absolute shrinkage and selection operator (Lasso) analysis (through the “glmnet” R package [29]) to avoid overfitting. Multivariate cox proportional hazard regression analysis was applied to construct the prognosis model. The risk scores of each of the patients were established with the score determined as: Risk score = ∑ (expression of signature genes * corresponding coefficient). According to the median risk score, patients from datasets were assigned to high-risk or low-risk groups.

### Prognostic signature validation

Datasets including internal and external validation sets were subsequently evaluated and used for calculating risk scores. The predictive capability of the signature was verified using ROC curves as well as KM survival curves (R packages “survival” and “survminer”). The individuals in the validation sets were allocated to groups by the same method as the training set. Furthermore, we also explored the predictive accuracy of nomograms through time-dependent ROC curves.

### Drug sensitivity analysis

To estimate the drug sensitivity in different risk groups, we performed drug sensitivity analysis utilizing the R package (“ggpubr”) and calculated the IC50 through the R package (“pRRophetic”) [30]. We therefore calculated the risk score of ccRCC cell lines through CCLE and drug sensitivity data in GDSC. Pearson correlation analysis was applied to identify the relationship between the risk score in ccRCC cell lines and the drug IC50 value.

### Immunotherapeutic effect analysis

Response to anti-PD-1/PD-L1 therapy in ccRCC patients was examined on three Immune-related cohorts (the Nivolumab group of the CheckMate 025 study, the Atezolizumab arm of IMmotion150 and the Atezolizumab plus Bevacizumab group of the IMmotion151 cohort). The comparison was different risk scores in the stable disease (SD), and progressive disease (PD) as well as complete response (CR) and partial response (PR) groups (R packages “ggsignif”, “ggplot2” and “ggpubr”). To predict the immunotherapeutic effect in different risk groups, the tumor immune dysfunction and exclusion (TIDE) (http://tide.dfci.harvard.edu/) was conducted and visualized through a violin plot.

### Enrichment analysis

Gene Ontology (GO) enrichment as well as Kyoto Encyclopedia of Genes and Genomes (KEGG) pathway analysis (R packages “clusterprofiler”) were utilized in this study and were further visualized by R package “ggplot2”. Gene set enrichment analysis (GSEA) was applied in this study and the “GSVA” R package was utilized to explore remarkably correlated pathways. Two annotated gene set file (“c2.cp.kegg.v7.4.symbols.gmt” as well as “c5.go.v7.5.1.symbols.gmt”) from the MSigDB database (https://www.gsea-msigdb.org/gsea/msigdb/) were chosen as the reference. ssGSEA was performed on each sample GSVA package as the input and computes an overexpression measure for a set of predefined gene sets of related pathways and immune checkpoints in the transcriptome. The ssGSEA scores were used in correlation analysis.

### Immunohistochemistry (IHC)

Paraffin sections of ccRCC tissues and normal tissues were deparaffinized and hydrated. Then, we performed antigen retrieval through microwave with citrate buffer (pH 9.0). To avoid the endogenous peroxidase activity, the sections were incubated in the slides in 0.3% H_2_O_2_ for 10 min. Then, sections were manipulated with primary antibody at 4 °C for 12 h and subsequently incubated with secondary antibody at room temperature for 1 h. After performing by peroxidase and 3,3-diaminobenzidinetetrahydrochloride (DAB), sections were developed with hematoxylin and subsequently mounted in nonaqueous mounting medium. Images were captured with KF-PRO-020 Digital Slice Scanner. Two qualified pathologists evaluated and scored pathological samples separately. The anti-IL4I1 antibody (1:200, Abcam, ab176588) and anti-CD206 (1:1000, Proteintech, China) were respectively utilized to detect expression levels of IL4I1 and CD206 in different tissues.

### Cell culture and siRNA interference assay

Human renal proximal tubular epithelial cell line (HK2) and Human RCC cell lines (786-O, 769-P, ACHN, A498, CAKI-1, CAKI-2 and OSRC2) sprang from the American Type Culture Collection (ATCC). All cells were maintained in appropriate medium with 10% FBS and incubated with 5% CO_2_ at 37 °C. One scrambled siRNA (negative control) and three IL4I1 siRNAs were synthesized by (RiboBio, China) (Additional file 3: Table S2). 786-O or 769-P was transfected with siRNAs through jetPRIME (Polyplus, French) on the basis of the manufacturer’s instructions. At 48 h after transfection, Functional assays were performed and protein and RNA were harvested.

### RNA expression

We extracted total RNAs from ccRCC cell lines and si-IL4I1-transfected 786-O or 769-P, as well as the ccRCC and adjacent normal samples of 10 KIRC patients using EZ-press RNA Purification Kit (EZBioscience, USA) and PrimeScript RT reagent kit (EZBioscience, China). The level of the mRNA IL4I1 was further examined through qRT-PCR with SYBR Green PCR reagent (EZBioscience, China) on Applied Biosystems™ QuantStudio™ 5 Real-Time PCR System in triplicate. Each mRNA expression was calculated with the 2-ΔΔCt method. All specific primers used in quantitative real-time PCR (qRT-PCR) are shown in Additional file 4: Table S3. Human participants in these studies were reviewed and approved by the Institutional Ethics Committee for Clinical Research and Animal Trials of the First Affiliated Hospital of Sun Yat-sen University [(2021)144] and conformed to the standards set by the Declaration of Helsinki.

### Wound healing, cell migration and invasion assays

Cells were scratched with a pipette after cellular fusion into a six-well plate. Photos were taken at 0 h and 24 h after scratching. To evaluate the invasion and migration capability, 786O or 769P were starved in serum-free RPMI 1640 medium for 8 h. Then, 5 × 10^4^ in 100 μl 786O or 769P in serum-free RPMI 1640 medium was added to transwell inserts (Corning, USA). The lower chamber of transwell assays was supplemented by serum-free RPMI 1640 medium with 10% FBS as a nutritional attractant. Lower surface cells after 8 h for migration assay and 16 h for invasion assay were respectively fixed with 4% polyformaldehyde (Beyotime, China) for 30 min, and stained with 0.4% crystalviolet (Beyotime, China) for 30 min. Invaded/migrated cells on the upper surface were wiped out with a cotton swab and those on the lower surface were counted under the microscope.

### Colony formation test

786-O or 769-P transfected with siRNAs were incubated in RPMI-1640 medium with 10% FBS, maintained in appropriate medium with 10% FBS and incubated with 5% CO_2_ at 37 °C. At 24 h after transfection, we inoculated 1000 786-O or 769-P cells into each well of the six-well plates, which were cultured in RPMI-1640 medium with 10% FBS for 2 weeks, then the colony was counted and analyzed.

### Coculture assay

THP-1 monocytes were induced in RPMI-1640 medium with 10% Fetal calf serum (FCS) and differentiated into M0 macrophages by 6 nM PMA (Phorbol 12-myristate 13-acetate). Once differentiated (M0 macrophages), they were incubated respectively with cell supernatant of ccRCC cell line (786-O or 769-P) transfected with IL4I1 siRNA for 48 h. The impact of the knock-down of IL4I1 on macrophage polarization was assessed by western blot for CD206 protein and flow cytometry of the proportion of M2 macrophage.

### Flow cytometry

Expression of M2 macrophage maker CD206 was examined through flow cytometry. The cells were detached with trypsin, washed and blocked with PBS + 1% BSA solution, and then incubated with CD206 (321104, Biolegend, California, USA). The cells were then analyzed by a Beckman CytoFlex LX instrument and analyzed by FlowJo software.

### Western blot

RIPA lysis buffer (ThermoFisher, USA) containing protease and phosphatase inhibitors (MCE) was utilized to extract total proteins incubated on ice for 15 min. Then, after centrifuged for 2 min (12,000×*g*, 4 °C) and supernatants collected, protein concentration was measured with a BCA protein assay kit (ThermoFisher, USA). After denaturation with 5× SDS-PAGE gels, the proteins were electrophoresed in 12% SDS-polyacrylamide gels (Bio-Rad) and transferred onto 0.2um PVDF membranes (Millipore) blocked in skim milk for 1 h. Then, membranes were incubated for 12 h with the primary antibody at 4 °C, and subsequently with the secondary antibody at room temperature for 1 h. Antibodies for western blots were as follows: IL4I1 (1:200, Abcam, ab176588), CD206 (1:1000, Proteintech, China).

### Statistical analysis

Pearson or Spearman coefficients were used to analyze correlations between variables. R language v4.0.2 (https://www.r-project.org/) and GraphPad Prism 8.0 software were conducted for data analysis. The evaluation of the difference between two groups was analyzed with the Wilcoxon rank-sum test. The significance of the two group differences was *P* < 0.05.

## Results

### Construction of the prognostic model

**Fig. 1:**
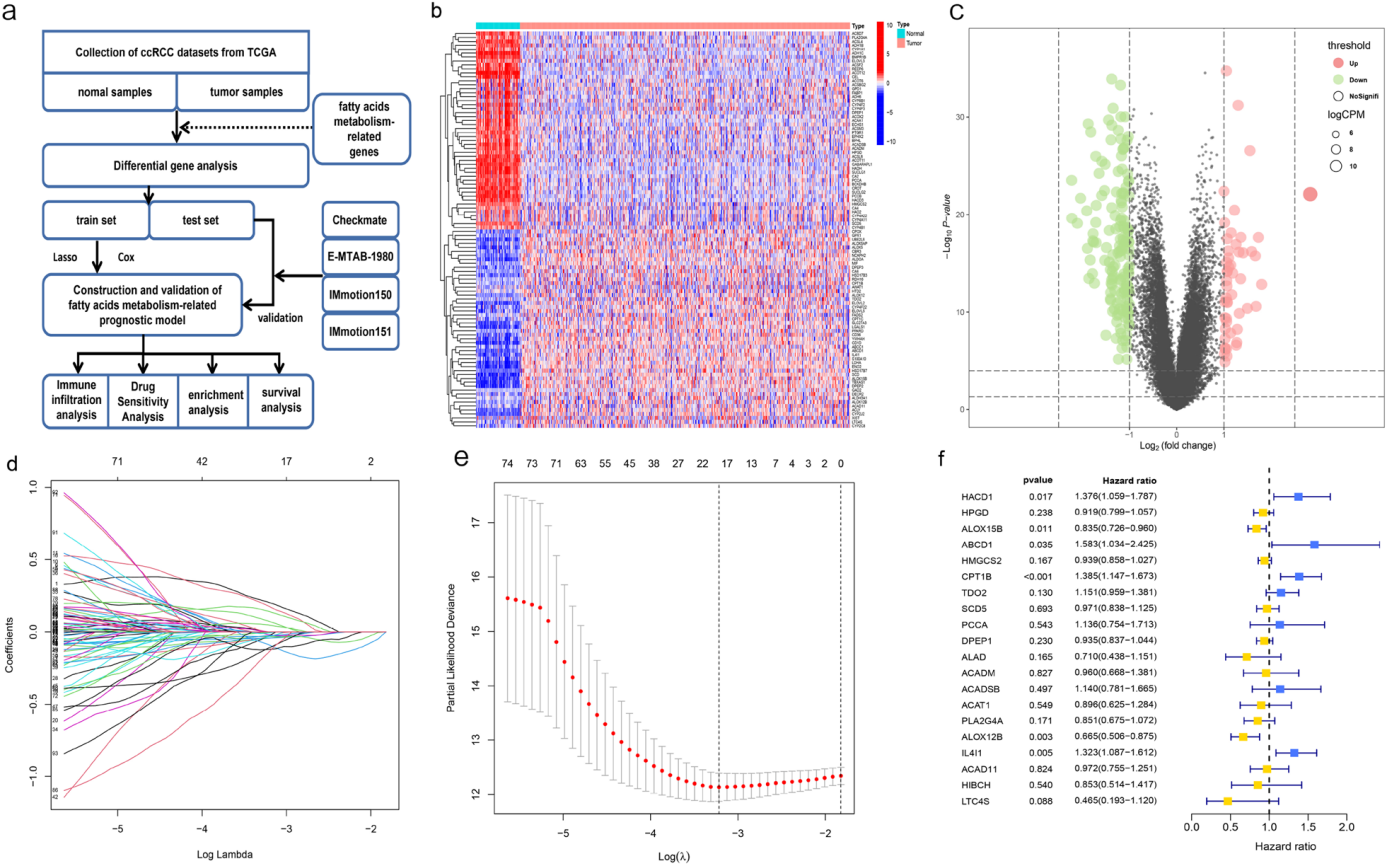
Overview of the study design and construction of the prognostic model. **a** Flow-chart of bioinformatics. **b** Heatmap of the differentially expressed fatty acids metabolism genes in KIRC. **c** Volcano plot of the downregulated and upregulated fatty acids metabolism-related DEGS. **d** Prognostic model construction by LASSO Cox analysis. **e** Cross-validation for the minimum lambda value in the LASSO regression model. **f** Forest plot of the prognostic model in multivariate cox analysis

A total of 309 genes related with fatty acids metabolism were included in this study. After TCGA datasets filtering, quality assessment and data processing, 96 DEGs were finally extracted through the “limma” R package and the results indicated that 62 DEGs were downregulated and 34 DEGs were upregulated. The heatmap and volcano map was used to visualize DEGs (Fig. 1b, c). The prognostic model was developed based on univariate and multivariate Cox regression analysis as well as LASSO analysis (Fig. 1d, e). Ultimately, the prognostic model was visualized by “forest” (Fig. 1f) consisting of 20 genes: HACD1, HPGD, ALOX15B, ABCD1, HMGCS2, CPT1B, TDO2, SCD5, PCCA, DPEP1, ALAD, ACADM, ACADSB, ACAT1, PLA2G4A, ALOX12B, IL4I1, ACAD11, HIBCH, LTC4S. Risk score = (0.319240 * HACD1) + (− 0.084391 * HPGD) + (− 0.180228 * ALOX15B) + (0.459381 * ABCD1) + (− 0.063391 * HMGCS2) + (0.325758 * CPT1B) + (0.140669 * TDO2) + (− 0.029671 * SCD5) + (0.127550 * PCCA) + (− 0.067707 * DPEP1) + (− 0.342020 * ALAD) + (− 0.040588 * ACADM) + (0.131207 * ACADSB) + (− 0.110134 * ACAT1) + (− 0.161595 * PLA2G4A) + (− 0.407284 * ALOX12B) + (0.280250 * IL4I1) + (− 0.028668 * ACAD11) + (− 0.158444 * HIBCH) + (− 0.766512 * LTC4S).

### Prognostic model validation

**Fig. 2:**
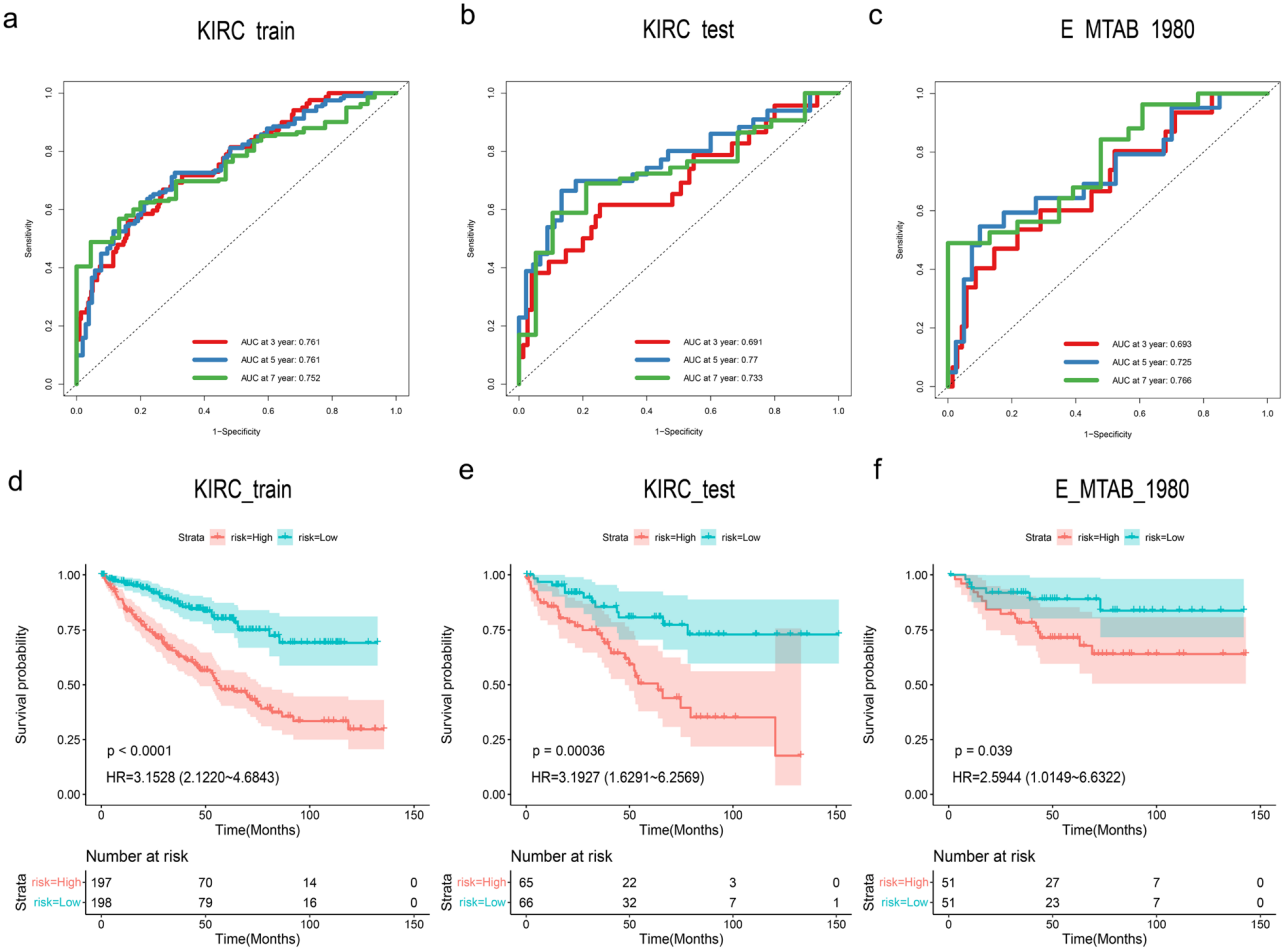
The predictive accuracy of the prognostic model. **a**–**c** ROC curves analysis of the 3-, 5-, and 7-year in TCGA-KIRC train and validation cohort as well as E-MTAB-1980 cohort respectively. **d**–**f** KM survival analysis of different risk groups in TCGA-KIRC train, validation cohort and E-MTAB-1980 respectively

The ROC curves and the KM curves were utilized to explore the predictive power of the prognostic model on the internal and external validation sets. The excellent results were found in the area under the ROC curve (AUC) of the 3-, 5-, and 7-year, with 0.761, 0.761, and 0.752 in the TCGA-KIRC train cohort; 0.691, 0.770, and 0.733 in the TCGA-KIRC validation cohort; and 0.693, 0.725, and 0.766 in the E-MTAB-1980 cohort (Fig. 2a–c). The results of AUCs greater than 0.68 in the train and validation cohort indicated that our model had a high sensitivity and specificity (high true positive rate and low false positive rate) and could accurately and robustly predict the prognosis of ccRCC patients. Using the same prognostic model, we classified the remaining patients in all the validation sets into different risk groups based on the median of all risk scores. The risk score of the prognostic model in TCGA-KIRC train and validation as well as E-MTAB-1980 cohort was an independent protective factor of overall survival (OS). Patients with high-risk scores exhibited significantly lower OS compared to those in the low-risk group (*P* < 0.05, Fig. 2d, e). K–M analysis further revealed that ccRCC patients with high-risk score had lower progression-free-survival (PFS) in TCGA-KIRC cohort (*P* < 0.05, Additional file 1: Figure S1a–d).

### The clinicopathological characteristics in TCGA-KIRC cohort

**Fig. 3:**
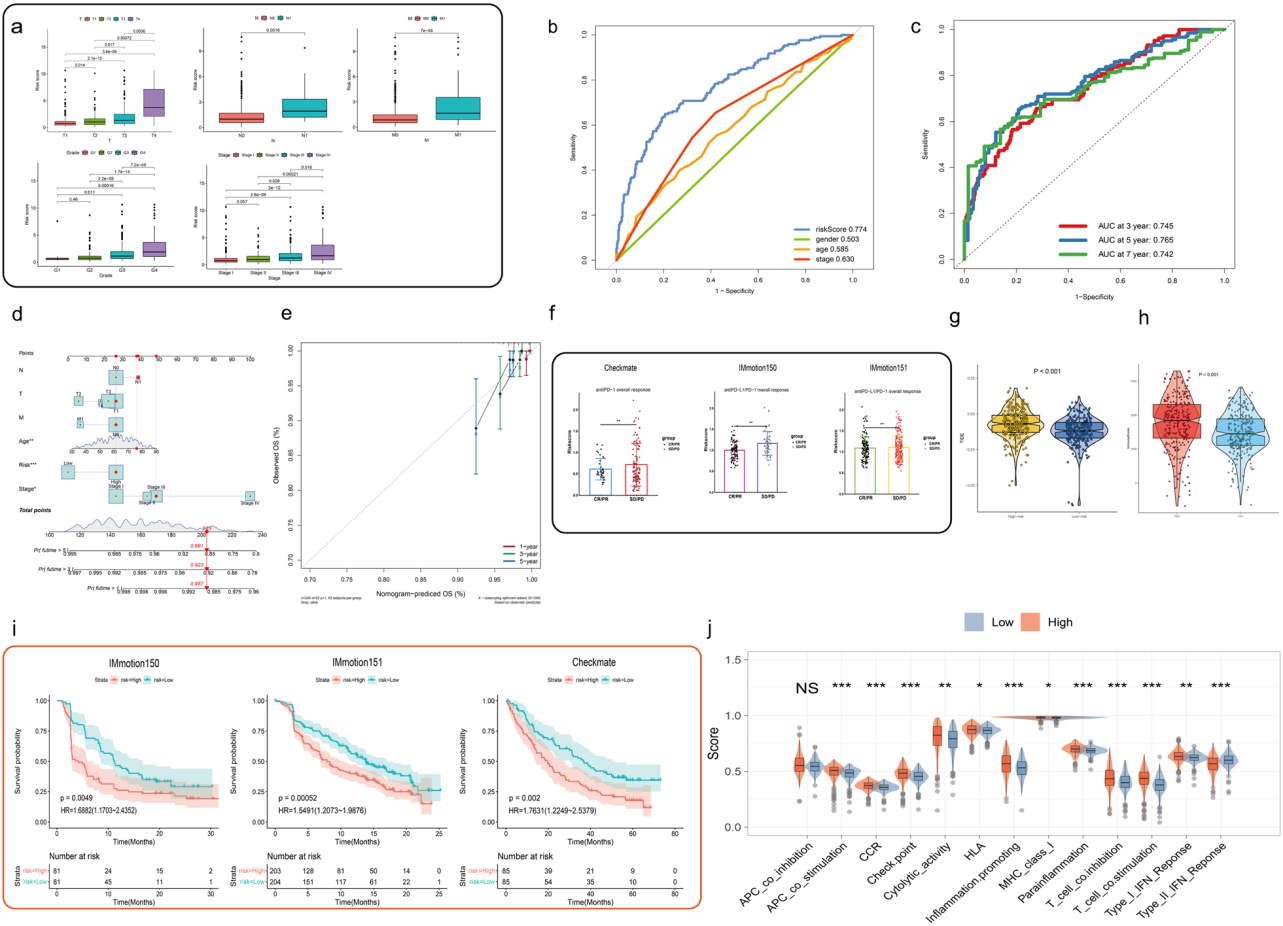
Clinical features and a new nomogram as well as immunological characteristics. **a** Clinical correlation analysis in different risk groups of TCGA-KIRC [T (Tumor), N (Node), M (Metastasis)]. **b** ROC curves analysis of risk score and clinical information in TCGA-KIRC cohort. **c** ROC curves of OS in TCGA-KIRC cohort at 3 year, 5 years, and 7 years. **d** The nomogram to predict 1-, 3-, and 5-year OS in TCGA-KIRC cohort based on risk scores and clinical factors. **e** The Calibration chart for the evaluation of nomogram accuracy. **f** Differences of risk scores between the stable disease (SD), and progressive disease (PD) as well as complete response (CR) and partial response (PR) in the antiPD-L1/PD-1 overall response. **g** The violin plot for TIDE score in different risk groups. **h** The comparison of immune scores in different risk groups. **i** Role of the risk score in the immune function of TCGA-KIRC cohort. **j** The comparison of several immune signatures in different risk groups

As shown in Fig. 3a, a clinical correlation analysis was performed, demonstrating that grade and stage levels increased with the risk score of the prognostic model (*P* < 0.05, Fig. 3a). Moreover, the risk score exhibited superior predictive power, with the largest area under the ROC curve compared to age, gender, grade, and stage (Fig. 3b). Furthermore, the AUC values of the 3-, 5-, and 7-year in TCGA-KIRC total cohort were 0.745, 0.765, and 0.742 respectively (Fig. 3c). Subsequently, based on the clinical features and risk score, a nomogram for OS prediction was conducted and composed of age, stage as well as risk score as the independent prognostic factors (*P* < 0.05) (see Fig. 3d). In the calibration diagram (Fig. 3e), the 1-, 3-, and 5-year OS for ccRCC individuals had a good predictive performance of this personalized nomogram model (C-index = 0.779).

### The immune landscape of the prognostic model and response to anti-PD-1/PD-L1 therapy

The response to anti-PD-1/PD-L1 therapy was examined based on the immune-related cohorts (the Nivolumab group of the CheckMate 025 study, the Atezolizumab arm of IMmotion150 and the Atezolizumab plus Bevacizumab group of the IMmotion151 cohort), and results indicated that different risk groups between the stable disease (SD), and progressive disease (PD) as well as complete response (CR) and partial response (PR) groups possessed a statistically significant difference. Not surprisingly, results from the immune-related databases revealed that the high-risk group processes a significantly lower response to anti-PD-1/PD-L1 therapy (Fig. 3f). Further, through the TIDE algorithm, high-risk group in the TCGA-KIRC database had a high potential of immune escape with a worse effect of immunotherapy (Fig. 3g). Applying the ESTIMATE algorithm, we calculated the overall levels of the immune score. The immune score of the high-risk group was higher than low-risk group (Figs. 3h). Moreover, as expected, in IMmotion150 and IMmotion151 cohorts, the low-risk group for survival was linked to higher PFS (*P* < 0.05, Fig. 3i). We then performed a hierarchically K-M analysis of the CheckMate-025 study. The results indicated there was a significant difference in the survival rate between different risk groups in the ccRCC patients with Nivolumab monotherapy from the CheckMate-025 cohort while there was no significant difference in those treated with Everolimus (a mammalian target of mTOR inhibitor) (Additional file 1: Fig. S1d, e), suggesting that our model is more effective and suitable for anti-PD-1/PD-L1 therapy in the ccRCC patients. Subsequently, in the comparison of several immune signatures, inflammation-promoting, T cell co-stimulation, checkpoint, antigen-presenting cell (APC) co-stimulation, chemokine receptors (CCR), and Type I IFN response were found to be higher in the high-risk group than the low-risk group, while type II IFN response was obviously downregulated (Fig. 3j). Additionally, TME cell composition and fraction of individual immune cell types in three Immune-related cohorts (the Nivolumab group of the CheckMate 025 study, the Atezolizumab arm of IMmotion150 and the Atezolizumab plus Bevacizumab group of the IMmotion151 cohort) were computed and generated with CIBERSORT (http://cibersort.stanford.edu/). We observed that the high-risk groups presented a higher fraction of M0 macrophages than the low-risk group (*P* < 0.05, Additional file 1: Fig. S2a–c), thus highlighting the risk score of our model could predict clinical response to ICI-based immunotherapy.

### Risk scores and drug sensitivity analysis

**Fig. 4:**
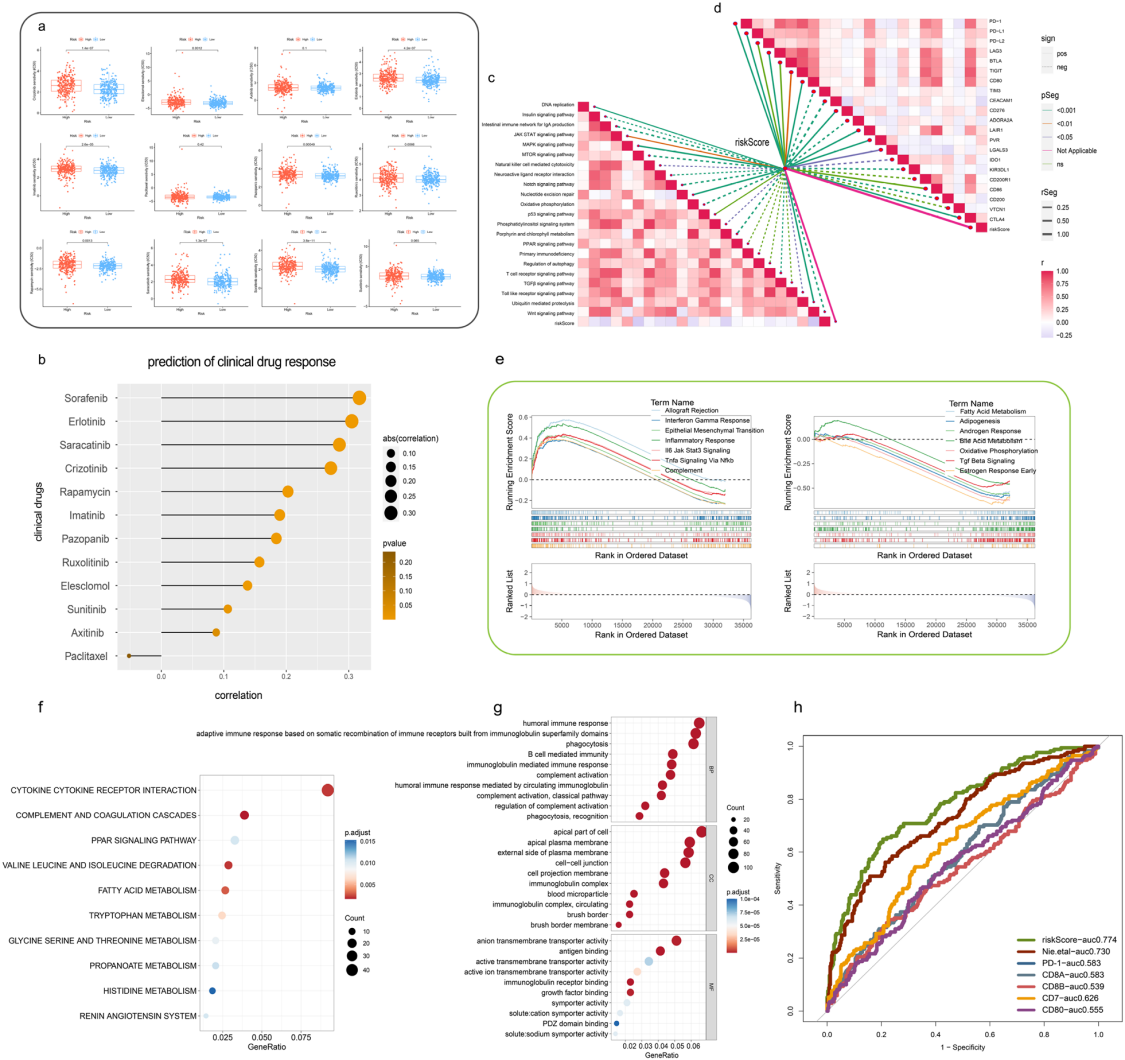
Drug sensitivity analysis and identifying related pathways. **a** Identification of drug sensitivity between high-risk group and low-risk group. **b** Correlation of drug targets and risk score in TCGA-KIRC cohort. **c** Pearson’s correlation analysis of functional pathways and risk score in TCGA-KIRC cohort. **d** Correlations between risk score and immune checkpoints. **e** Comparison of the enrichment of functional pathways by GSEA analysis of different related risk score. **f** KEGG enrichment of DEGs. **g** GO enrichment analysis of the top 30 pathways in different risk groups. **h** The ROC curve of joint indicators and multi-model comparison in TCGA-KIRC cohort

To predict clinical response to targeted therapy for high-risk ccRCC patients, we screened chemotherapy drugs in the treatment of ccRCC patients We screened and estimated chemotherapy drugs in ccRCC based on the different risk groups through the “pRRophetic” package and the IC50 value was utilized to measure the sensitivity to drugs (Fig. 4a). The high-risk group possessed significantly higher IC50 values but was less sensitive to the drugs than the group with low-risk (*P* < 0.05) (Fig. 4b). For quantifying the individual patients, the correlations between risk score of prognostic model and targeted drugs was assessed through the “ggplot” package and identified that Sorafenib, Erlotinib, Saracatinib and Crizotinib had high degree of correlation with risk score of prognostic model, suggesting that the constructed model could effectively predict efficacy and sensitivity to chemotherapy and aid in clinical feasibility of drug screening-guided precision therapy of ccRCC. To identify useful therapeutic implications and design of effective fatty-acids-metabolism targeted therapy, we further evaluated the risk score of ccRCC cell lines through CCLE and drug sensitivity data from GDSC. The results indicated that A498 had the highest risk score while BFTC-909 had the lowest risk score, which implied that higher malignancy existed in A498 compared with other cell lines (Additional file 1: Fig. S3a). Pearson’s correlation analysis suggested that the risk score had a positive correlation with IC50 of C-75 (an inhibitor of fatty acids synthase) (Additional file 1: Fig. S3b). These revealed that the higher risk score was less sensitive to C-75, which might be a potential corresponding compound for treatment to low-risk ccRCC patients.

### Identifying related pathways and immune checkpoints

We utilized ssGSEA to examine the correlations between the risk score and the enrichment scores of related pathways or immune checkpoints to explore the immune-related functional processes. Our findings revealed a positive correlation between the risk score and JAK-STAT3 signaling, as well as major immune checkpoints (Fig. 4c, d). These implied that fatty acids metabolism in ccRCC was associated with JAK-STAT3 signaling and the risk score levels could reflect the therapeutic effect of ccRCC patients treated with anti-PD-1/PD-L1.

### Enrichment analysis and GSEA hallmark visualization

To confirm the underlying mechanism of JAK-STAT3 signaling and fatty acids metabolism in ccRCC progression, we performed GSEA on TCGA-KIRC cohort. The results based on GSEA analysis indicated that differentially expressed target genes are enriched in immune and metabolism-related functional pathways. The upregulated DEGs groups, in particular, were enriched in JAK-STAT3 signaling. Meanwhile, fatty acids metabolism was observed in the downregulated DEGs groups (see Fig. 4e). KEGG enrichment analysis was developed for tumorigenic pathways enrichment analysis in TCGA-KIRC cohort which demonstrated an association with cytokine-cytokine receptor interaction, fatty acids metabolism and complement and coagulation cascades (Fig. 4f). GO analysis was performed to further explore the potential biological processes of DEGs and revealed that most immune responses and associated activities were significantly enriched in these genes (Fig. 4g). Thus, these results indicated that the fatty acids metabolism might contribute to ccRCC development mainly concentrated on immune response.

### Evaluation of joint indicators and multi model comparison

To establish the superiority of our model, we conducted a comparison of the accuracy of several immune indicators and previous studies in the TCGA-KIRC cohort [31]. The ROC curve indicated that the AUC of the risk score is significantly higher than other indicators and other fatty acids metabolism-related model, suggesting that our model was more accurate (Fig. 4h, Additional file 1: Fig. S4). Overall, the constructed model demonstrated greater representativeness in fatty acids metabolism compared to other network models or indicators.

### Validation of mRNA expression

**Fig. 5:**
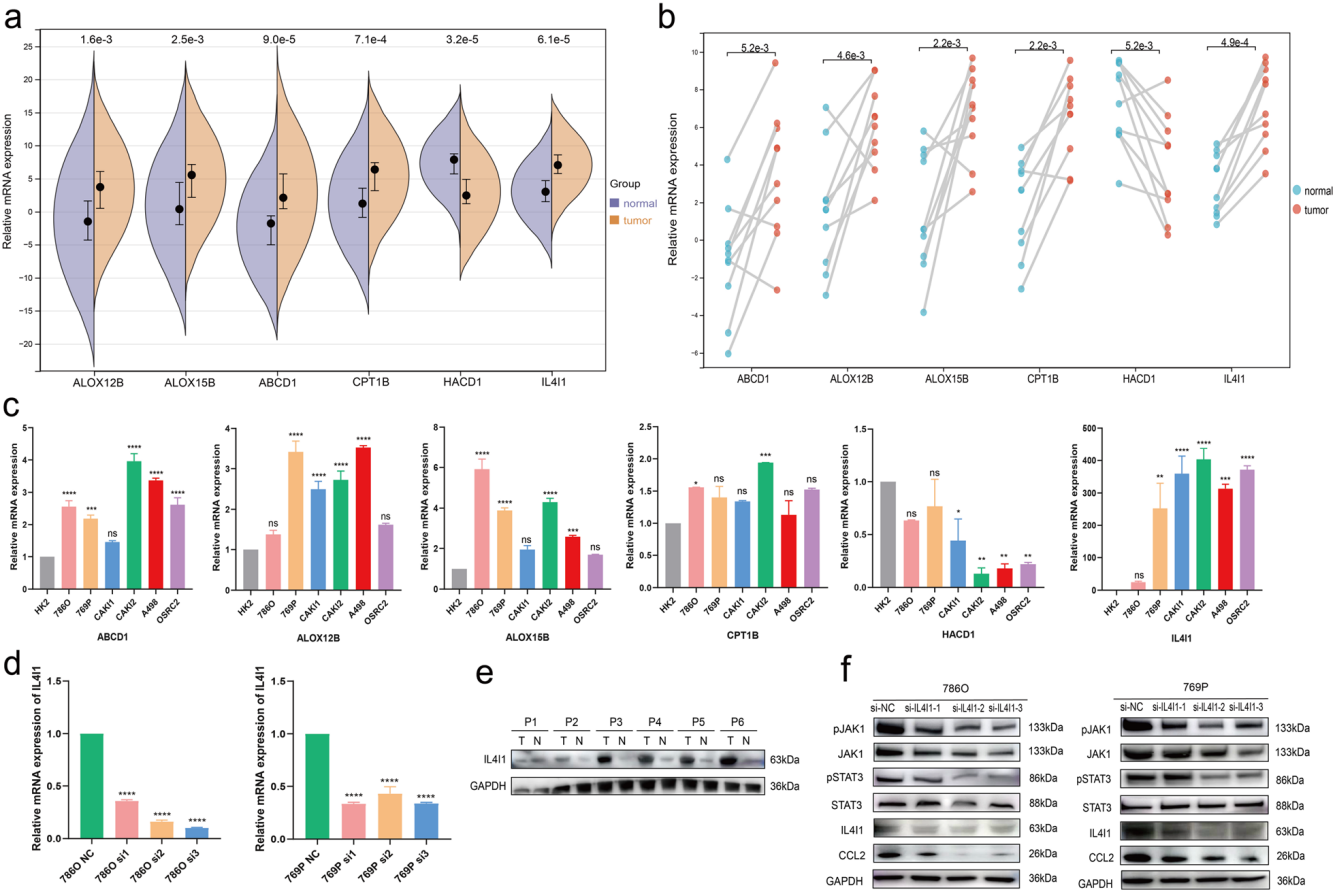
Prediction and verification of expression levels of mRNA and protein. **a** mRNA expression of ABCD1, ALOX12B, ALOX15B, CPT1B, HACD1 and IL4I1 in normal as well as tumor samples. **b** mRNA expression of ABCD1, ALOX12B, ALOX15B, CPT1B, HACD1 and IL4I1 in 10 pairs normal and tumor samples through matched-sample comparison. **c** mRNA expression of ABCD1, ALOX12B, ALOX15B, CPT1B, HACD1 and IL4I1 in different cell lines. **d** Levels of the mRNA expression in si-IL4I1-transfected ccRCC cells (786-O and 769-P). **e** Protein expression of IL4I1 in ccRCC tumor tissue and normal tissues. **f** Protein expression of IL4I1, JAK1, pJAK1, STAT3, pSTAT3, and CCL2 in si-IL4I1-transfected ccRCC cells (786-O and 769-P). *P < 0.05; **P < 0.01; ***P < 0.001; ****P < 0.001

To analyze the mRNA expression profiles, we explored the expression levels of 6 fatty acids metabolism-related genes in our risk model in tumor and normal tissues as well as ccRCC cell lines. The qPCR results indicated that the expression levels of ABCD1, ALOX12B, ALOX15B, CPT1B, IL4I1 significantly upregulated in tumor samples, while the expression of HACD1 was low in ccRCC tissues (Fig. 5a, b). We also identified these genes expression in TCGA-KIRC cohort and the result indicated the tendency of significant genes expressed the similar (Additional file 1: Fig. S5). Consistently, the expression patterns of these six genes were also observed in ccRCC cell lines (Fig. 5c). Additionally, oligo sequences in the qPCR were displayed in Additional file 3: Table S2.

### Silencing IL4I1 suppresses the growth and invasion of ccRCC cells

**Fig. 6:**
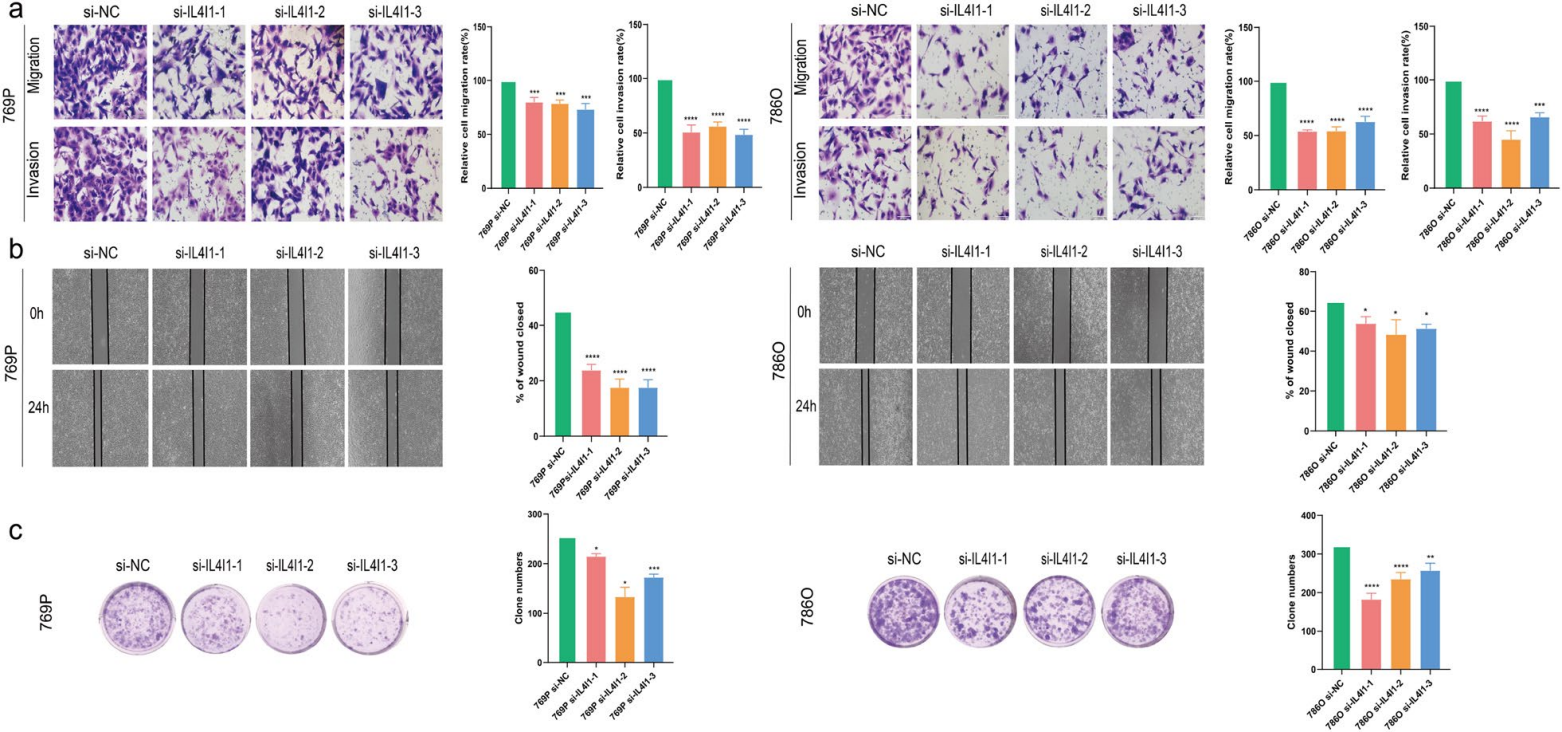
Verification of IL4I1 for proliferation, migration, and invasion in ccRCC. **a** Transwell migration/invasion assay to analyse migration and invasion of ccRCC cell. **b**, **c** Would healing and colony formation assays to detect ccRCC cell proliferation. *P < 0.05; **P < 0.01; ***P < 0.001; ****P < 0.001

To elucidate the role of IL4I1 in ccRCC migration and invasion in vitro, three siRNA specifically targeting IL4I1 (si-IL4I1-1, si-IL4I1-2 and si-IL4I1-3) were constructed respectively (Fig. 5d). Transwell assays revealed that the migration and invasion capabilities of 786-O and 769-P were inhibited after the silence of IL4I1 (Fig. 6a). Furthermore, in accordance with the above results, colony formation assays and wound healing demonstrated that silencing IL4I1 significantly suppresses the proliferation of 786-O and 769-P (Fig. 6b, c). Altogether these results collectively elucidated that IL4I1 could promote the growth and invasion of ccRCC cells.

### Silencing IL4I1 impacts JAK1/STAT3 signaling pathway

Based on the above analysis about our model, GSEA suggested that our model strongly associated with JAK1/STAT3 signaling pathway (Fig. 5e). To further clarify the effects of IL4I1 on JAK1/STAT3 signaling pathway, western blot was assessed to test IL4I1’s contribution to the functional outcomes of JAK1/STAT3 signaling pathway. The result showed that silencing IL4I1 downgrades the expression of phosphorylated JAK1 and phosphorylated STAT3 (Fig. 5f), suggesting that IL4I1 could modulate JAK1/STAT3 signaling pathway and lead to JAK1/STAT3 phosphorylation.

### Silencing IL4I1 inhibits M2-like macrophages polarization

**Fig. 7:**
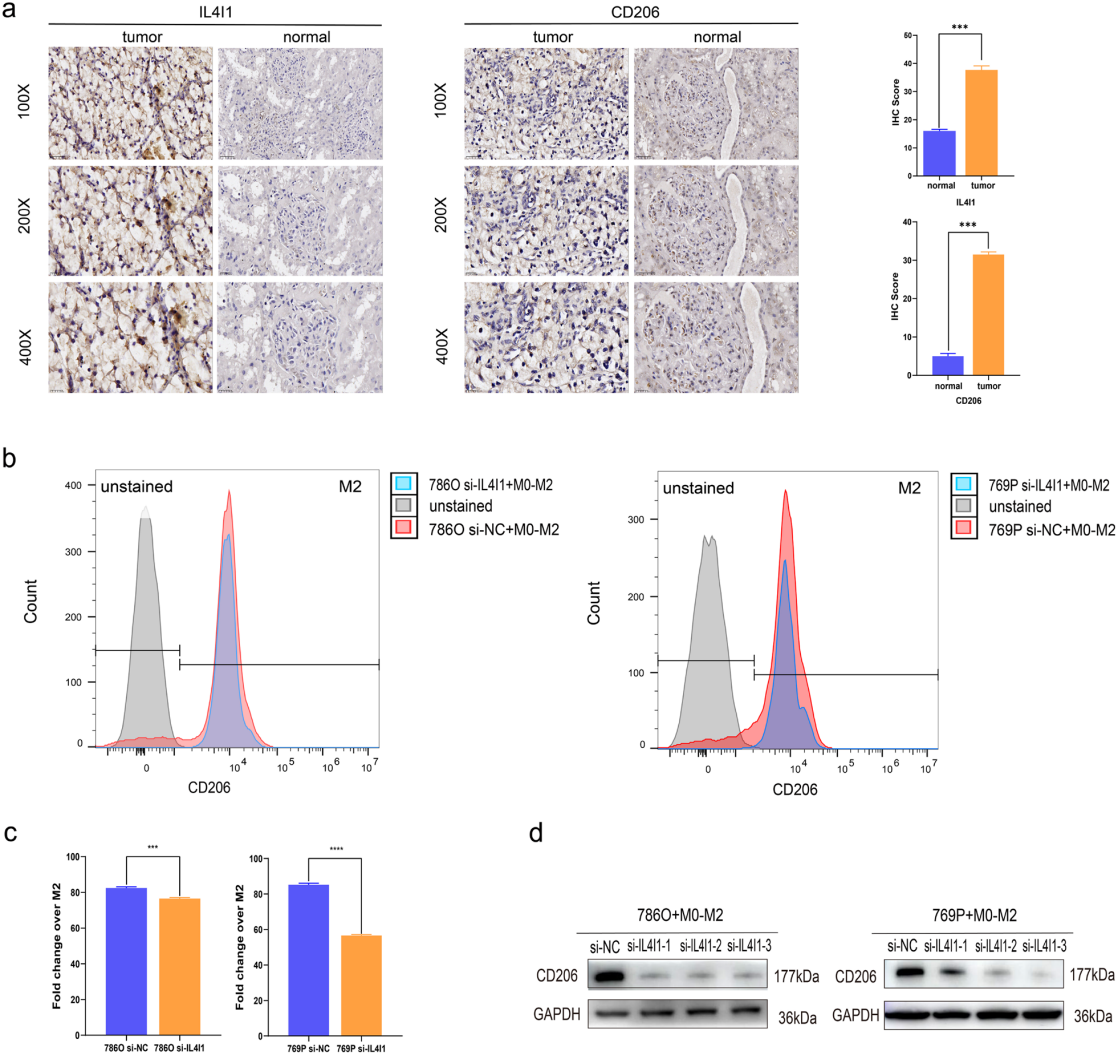
Immunohistochemistry and Coculture assay. **a** Expression of IL4I1and CD206 in ccRCC tumor tissue and normal tissues by IHC. **b**, **c** Flow cytometry to detect M2-like macrophage polarization. **d** M2-like macrophage polarization after coculture assay conducted by western blot. *P < 0.05; **P < 0.01; ***P < 0.001; ****P < 0.001

To investigate whether IL4I1 signaling to CCL2 could mediate macrophages polarization, an indirect co-culture condition between si-IL4I1-transfected 786-O and 769-P as well as M0-like macrophages was conducted. Flow cytometry results revealed that silencing IL4I1 inhibited M2-like macrophages polarization (Fig. 7b, c). Additionally, western blot also implied that silencing IL4I1 could suppress the level of CD206 (M2 macrophage surface marker) (Fig. 7d) and even CCL2 expression of si-IL4I1-transfected ccRCC cells (786-O and 769-P) (Fig. 5f). Moreover, IHC staining validated that the high expression of IL4I1 and CD206 in tumor samples were simultaneously higher than that in adjacent normal tissues (Fig. 7a). In summary, these results proved that the knockdown of IL4I1 in si-IL4I1-transfected 786-O and 769-P inhibited M2-like macrophage polarization engaged by the regulation of CCL2.

## Discussion

The dynamic fatty acids metabolism disorder of malignant cells has a profound influence on tumor-targeting immune responses in TME. Dysregulated fatty acids metabolism can lead to the accumulation of lipids, which in turn can modulate the activity of TAMs and suppress immune surveillance [12, 32]. In particular, high expression of fatty acids synthase (FAS) serves as an adverse predictive marker for survival prognosis [33]. CCL2 can stimulate and mediate the infiltration and migration of macrophages to the inflamed site and induce M2 phenotype polarization in TME to accelerate carcinogenesis [34]. CCL2 and its receptor CCR2 are strongly associated with Crosstalk between fatty acids metabolism and TAMs would offer a novel treatment strategy to ccRCC. While a previous report has constructed a prognosis model for fatty acids metabolism in ccRCC [31], it lacks satisfactory predictive abilities, and is unable to associate TME with fatty acids metabolism through a well-defined method, lacking the corresponding immune validation set and experimental evidence.

In the study, we constructed a fatty acids metabolism-related prognosis model and explored mechanisms that fatty acids metabolism impacts on the therapeutic effect of anti-PD-1/PD-L1 in TME in ccRCC. Based on related fatty-acids-metabolism-related genes in TCGA-KIRC train cohort, an efficient prognostic model for ccRCC patients consisting of 20 genes was constructed through univariate and multivariate Cox regression analysis as well as LASSO analysis. We further explore the prognostic value of model on the internal and external validation sets through ROC curves and KM curves by the median of all risk scores. The AUC of the TCGA-KIRC train, and the validation cohort as well as E-MTAB-1980 cohort demonstrated a good prognostic efficiency. Additionally, ccRCC patients with high-risk score in all cohorts had significantly lower survival probability. High-risk groups were found to be correlated with higher tumor grade and advanced pathologic stage in the TCGA-KIRC cohort. Subsequently, a new nomogram was conducted including independent prognostic factors (age, stage, and risk score) and had a good predictive performance. For the purpose of promotion in immunotherapy in ccRCC patients, based on three Immune-related cohorts (the Nivolumab group of the CheckMate 025 study, the Atezolizumab arm of IMmotion150 and the Atezolizumab plus Bevacizumab group of the IMmotion151 cohort), results indicated that high-risk group had a significantly lower response to anti-PD-1/PD-L1 therapy. Further investigation revealed that a high potential of immune escape phenotype existed in the high-risk group in the TCGA-KIRC cohort through the TIDE algorithm, implying that high risk score in this model predicts poor prognosis in ccRCC patients treated with immunotherapy. In addition, we also found that the higher immune score occurred in the high-risk group. Therefore, our findings provide a model to identify which type of ccRCC patients are more suitable for immunotherapy and achieve a better curative effect. ccRCC is more frequently known as a proinflammatory neoplasia and can recruit polyclonal CD8^+^T cells through cytokines production [35–38]. However, high densities of CD8^+^T cells are involved in the poor clinical prognosis of ccRCC patients [39]. Consistent with these findings, immune signatures analysis in TCGA-KIRC cohort suggested that inflammation-promoting, T cell co-stimulation, checkpoint, CCR, and Type I IFN response were found to be higher in the high-risk group than the low-risk group. Our findings also observed that the high-risk group presented significantly higher IC50 values and was less sensitive to the agents than the low-risk group. To aid in clinical decision-making, drug sensitivity, Pearson correlation analysis were performed and indicated that the risk score had positive correlation with IC50 of C-75. Interestingly, C-75 is an inhibitor of FAS which triggers apoptosis during S phase and inhibits fatty acids synthesis in liver cancer [40, 41], which might be an effective targeted therapy of ccRCC. To analyze if the model could provide new indications for immunotherapy, it was noteworthy that the related functional pathways or immune checkpoints were recognized to be positively correlated with IL6-JAK-STAT3 signaling and prominent immune checkpoints (including PD-1, PD-L1, LAG-3, and CTLA4). We compared the accuracy of several associated immune indicators and previous studies in the TCGA-KIRC cohort and found that the risk score had a significantly higher AUC than other indicators and previous models related to fatty acids metabolism. Among the 20 genes in the model, we selected genes with a p-value threshold of less than 0.05 (including ABCD1, ALOX12B, ALOX15B, HACD1, IL4I1, CPT1B) in prognostic model for sequent analysis. Furthermore, we performed RT-qPCR to consistently evaluated the expression levels of ABCD1, ALOX12B, ALOX15B, HACD1, IL4I1, CPT1B by RT-qPCR. Abnormal gene expression patterns emerged into these 6 genes which might be closely related to the tumorigenesis of ccRCC. Of them, ATP-binding-cassette transporter subfamily D member 1 (ABCD1) transports very long chain fatty acyl-CoAs from cytosol to peroxisome for β-oxidation [42]. Reductive levels of ABCD1 in ccRCC inhibit tumor migration and tumor sphere formation. ccRCC patients with high ABCD1 expression were associated with reduced overall survival [43]. Arachidonate 12-lipoxygenase, 12R type (ALOX12B) can convert arachidonic acids to 12R-hydroxyeicosatetraenoic acids 8 and is responsible for immune activity blocking the uptake of apoptotic cells through inflammatory monocytes which reduces antigen presentation to T cells in tumor [44]. Downregulation of ALOX12B prompts poor survival rate and advanced stages negative HPV 16-negative head and neck squamous cell carcinoma [45]. Arachidonate 15-lipoxygenase, type B (ALOX15B) upregulated in RCC-infiltrating macrophages, mediates lipid metabolism in TAMs and contributes to tumor progression as well as tumor immunity [46, 47]. ALOX15B was significantly downregulated in bladder cancer tissues and ALOX15B downregulation could promote bladder cancer cell motility [48]. 3-Hydroxyacyl-CoA dehydratase 1 (HACD1) has been implicated as a regulator in the membrane composition and fluidity and elongate the very long chain fatty acids [49]. Carnitine palmitoyltransferase 1B (CPT1B) exerts rate-controlling-enzyme roles in fatty acids β-oxidation, could be inhibited by inhibiting JAK/STAT3 promoting breast cancer cells to re-sensitize to chemotherapy [50]. Upregulation of CPT1B is correlated with poor prognosis in prostate cancer and overexpression of CPT1B promotes the resistance of C4-2R cells to enzalutamide [51]. More importantly, Interleukin-4-induced-1 (IL4I1) as a metabolic immune checkpoint, activates the Aryl hydrocarbon receptor (AHR), circumvents Immune Checkpoint Blockade (ICB) and further elicits major effects in immunosuppression shaping tumor microenvironment [52]. IL4I1 downregulation reduces inflammatory mediators and restrains the accumulation of triglyceride and palmitate (a 16-carbon long fatty acid) to inhibit inflammatory response via AHR activation [53]. Moreover, previous study indicated that IL4I1 regulated and enhanced M2 macrophage polarization through the phosphorylation of STAT-6 and STAT-3 to suppress T cell activation [54]. More importantly, advanced ccRCC is featured as terminally exhausted CD8 T cells and M2-like macrophages simultaneously and expresses ligands and receptors to support T cell dysfunction and M2-like polarization [11]. The above observations highlight that IL4I1 processes promote effects on ccRCC occurrence, and pleaded for scrutiny in the role of IL4I1 as a prognosis factor in the crosstalk of fatty acids metabolic reprogramming and immune regulatory functions in ccRCC. Simultaneously, we noted that the proliferation, migration and invasion capability of ccRCC cells silencing IL4I1 were decreased. Beyond its correlations with immune processes, the results based on GSEA analysis revealed that IL4I1 was enriched in the following activated pathways, containing IL6-JAK-STAT3 signaling. GO functional enrichment analysis exhibited that most immune responses and associated activities were mainly enriched in these genes. KEGG indicated its association with fatty acids metabolism. To further clarify the effects of IL4I1 on JAK1/STAT3 signaling pathway, western blot was assessed to test IL4I1’s contributions to the functional outcomes of JAK1/STAT3 signaling pathway. Together, these findings implied that IL4I1 could modulate JAK1/STAT3 signaling pathway and lead to JAK1/STAT3 phosphorylation. To explore if IL4I1 signaling to CCL2 could mediate macrophages polarization, an indirect co-culture condition between si-IL4I1-transfected ccRCC cells (786-O and 769-P) and M0-like macrophages was conducted and revealed that silencing IL4I1 inhibited M2-like macrophages polarization associated with JAK1/STAT3 phosphorylation and downregulated CCL2 expression. Taken overall, IL4I1 might activate CCL2-CCR2 via JAK1/STAT3 signaling pathway further to promote M2-like macrophages polarization which enhances ccRCC immune escape and malignant behaviors.

To summarize, we describe a novel mechanism underlying fatty acids metabolism that promotes the crosstalk within and across cancer cells and immune cells in ccRCC tumor microenvironment. However, several limitations still exist. Firstly, the optimal cut-off value of risk score was not determined and the median risk score was used as a surrogate. Furthermore, additional in vivo and in vitro experiments are required to further elucidate the comprehensive role of the fatty acids metabolism-related prognostic model in ccRCC. However, this study represents the most systematical and comprehensive investigation that elucidates how the fatty acids metabolism influences resistance to immunotherapy in ccRCC, which could serve as potentially a potent orientation to evaluate low immune responses in ccRCC patients.

## Conclusion

This study demonstrated that fatty acids metabolism affects alternative polarization of immune cells correlated with JAK1/STAT3 signaling pathway and CCL2, further influencing the therapeutic effect of PD-1/PD-L1 to ccRCC in TME.

## Supplementary Information

**Additional file 1: Figure S1.** K–M analysis of different-risk groups. (a–c) K–M analysis of PFS of different-risk groups in TCGA-KIRC train, validation, total cohort. (d, e) K–M analysis of different-risk groups of OS in Checkmate-total and everolimus cohort. **Figure S2.** TME cell composition and fraction of individual immune cell types in three Immune-related cohorts. (a) the Nivolumab group of the CheckMate 025 study. (b) the Atezolizumab arm of IMmotion150. (c) the Atezolizumab plus Bevacizumab group of the IMmotion151 cohort. **Figure S3.** The risk score of ccRCC cell lines through CCLE and drug sensitivity data in GDSC. (a) A498 had the highest risk score while BFTC-909 had the lowest risk score. (b) The risk score had positive correlation with IC50 of C-75. **Figure S4.** (a–d) The ROC curve of joint indicators and multi-model comparison in TCGA-KIRC cohort in E-MTAB-1980, the Nivolumab group of the CheckMate 025 study, the Atezolizumab arm of IMmotion150 and the Atezolizumab plus Bevacizumab group of IMmotion151 cohort. **Figure S5.** (a) mRNA expression of ABCD1, ALOX12B, ALOX15B, CPT1B, HACD1 and IL4I1 in TCGA-KIRC cohort.

**Additional file 2.** Fatty acids metabolism-related genes.

**Additional file 3.** Three IL4I1 siRNAs sequence.

**Additional file 4.** All primers sequence.

## Data Availability

Any reasonable requests for access to available data underlying the results reported in this article will be considered. Such proposals should be submitted to the corresponding author.

## Abbreviations

RCC: Renal cell carcinoma
ccRCC: Clear cell renal cell carcinoma
ICTs: Immunocheckpoint therapies
TME: Tumor microenvironment
PD-1: Programmed death-1 receptor
PD-L1: Programmed death-1 receptor-ligand
DEG: Differentially expressed gene
TIDE: Tumor immune dysfunction and exclusion
FC: Fold change
FDR: False Discovery Rate
Lasso: Least absolute shrinkage and selection operator
SD: Stable disease
PD: Progressive disease
CR: Complete response
PR: Partial response
GSEA: Gene set enrichment analysis
ssGSEA: Single sample Gene set enrichment analysis
GSVA: Gene set variation analysis
KEGG: Kyoto encyclopedia of genes and genomes
KIRC: Kidney renal clear cell carcinoma
TCGA: The Cancer Genome Atlas
FAS: Fatty acids synthase
CCL2: C-C motif chemokine ligand 2
CCR2: C-C chemokine receptor type 2
TAMs: Tumor-associated macrophages
CCLE: Cancer Cell Line Encyclopedia
GDSC: Genomics of Drug Sensitivity in Cancer
AHR: Aryl hydrocarbon receptor
ICB: Immune Checkpoint Blockade
OS: Overall survival
PFS: Progression free survival
DAB: 3,3-Diaminobenzidinetetrahydrochloride
PMA: Phorbol 12-myristate 13-acetate
IL4I1: Interleukin-4-induced-1
CPT1B: Carnitine palmitoyltransferase 1B
ABCD1: ATP-binding-cassette transporter subfamily D member 1
ALOX12B: Arachidonate 12-lipoxygenase, 12R type
ALOX15B: Arachidonate 15-lipoxygenase, type B
HACD1: 3-Hydroxyacyl-CoA dehydratase 1
HPGD: 15-Hydroxyprostaglandin dehydrogenase
HMGCS2: 3-Hydroxy-3-methylglutaryl-CoA synthase 2
TDO2: Tryptophan 2,3-dioxygenase
SCD5: Stearoyl-CoA desaturase 5
PCCA: Propionyl-CoA carboxylase subunit alpha
DPEP1: Dipeptidase 1
ALAD: Aminolevulinate dehydratase
ACADM: Acyl-CoA dehydrogenase medium chain
ACADSB: Acyl-CoA dehydrogenase short/branched chain
ACAT1: Acetyl-CoA acetyltransferase 1
PLA2G4A: Phospholipase A2 group IVA
ACAD11: Acyl-CoA dehydrogenase family member 11
HIBCH: 3-Hydroxyisobutyryl-CoA hydrolase
LTC4S: Leukotriene C4 synthase
JAK/STAT3: Janus tyrosine kinase1/signal transducer and activator of Transcription3
