# Supplementary material for: Fatty acids metabolism affects the therapeutic effect of anti-PD-1/PD-L1 in tumor immune microenvironment in clear cell renal cell carcinoma
Source: J Transl Med. 2023 May 23;21:343. doi: 10.1186/s12967-023-04161-z (PMC10204332; doi:10.1186/s12967-023-04161-z)
Supplement: Supplementary file 1 — Additional file 1: Figure S1. K–M analysis of different-risk groups. (a–c) K–M analysis of PFS of different-risk groups in TCGA-KIRC train, validation, total cohort. (d, e) K–M analysis of different-risk groups of OS in Checkmate-total and everolimus cohort. Figure S2. TME cell composition and fraction of individual immune cell types in three Immune-related cohorts. (a) the Nivolumab group of the CheckMate 025 study. (b) the Atezolizumab arm of IMmotion150. (c) the Atezolizumab plus Bevacizumab group of the IMmotion151 cohort. Figure S3. The risk score of ccRCC cell lines through CCLE and drug sensitivity data in GDSC. (a) A498 had the highest risk score while BFTC-909 had the lowest risk score. (b) The risk score had positive correlation with IC50 of C-75. Figure S4. (a–d) The ROC curve of joint indicators and multi-model comparison in TCGA-KIRC cohort in E-MTAB-1980, the Nivolumab group of the CheckMate 025 study, the Atezolizumab arm of IMmotion150 and the Atezolizumab plus Bevacizumab group of IMmotion151 cohort. Figure S5. (a) mRNA expression of ABCD1, ALOX12B, ALOX15B, CPT1B, HACD1 and IL4I1 in TCGA-KIRC cohort. [file 12967_2023_4161_MOESM1_ESM.docx]

**Additional file 1**

**
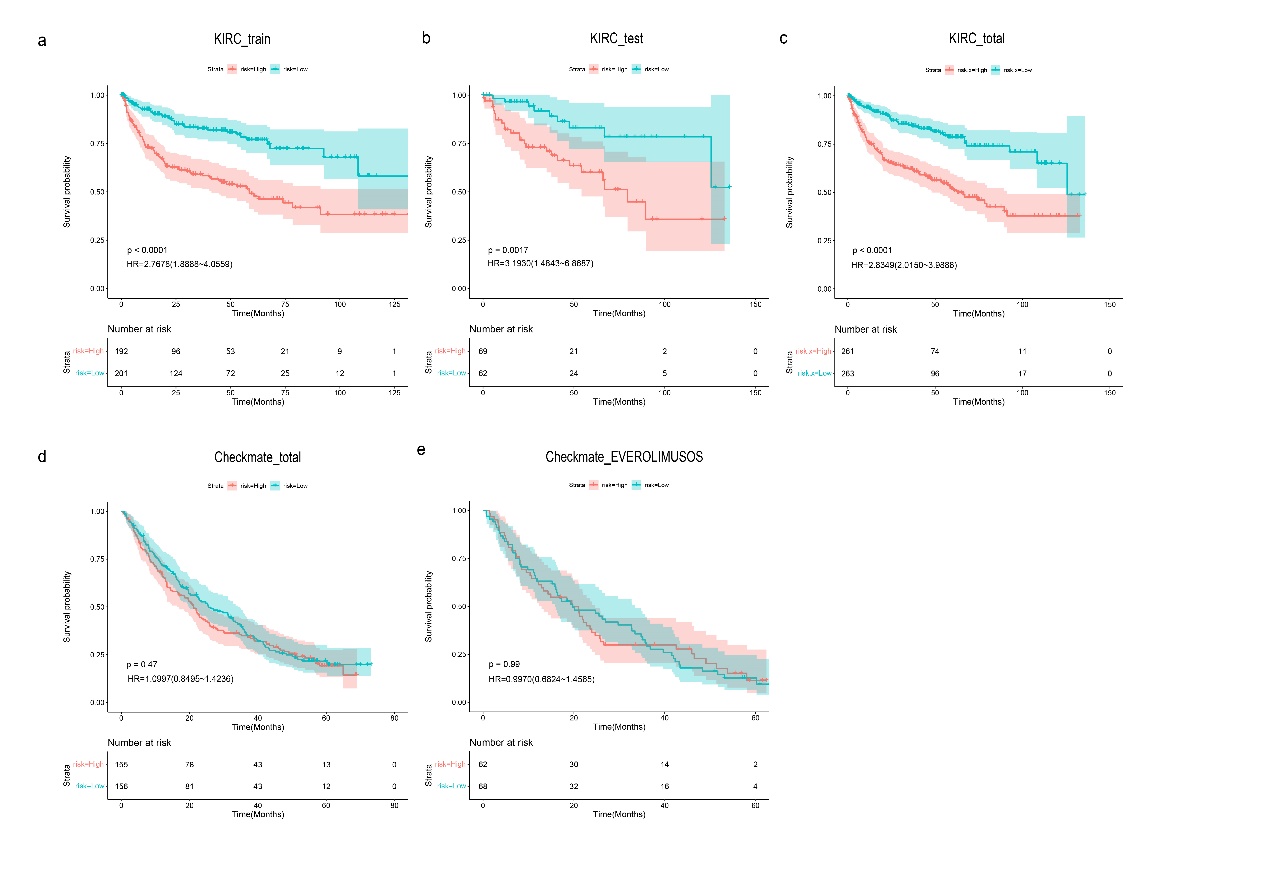
**

Figure S1. K-M analysis of different-risk groups. (a-c) K-M analysis of PFS of different-risk groups in TCGA-KIRC train, validation, total cohort. (d-e) K-M analysis of different-risk groups of OS in Checkmate-total and everolimus cohort.


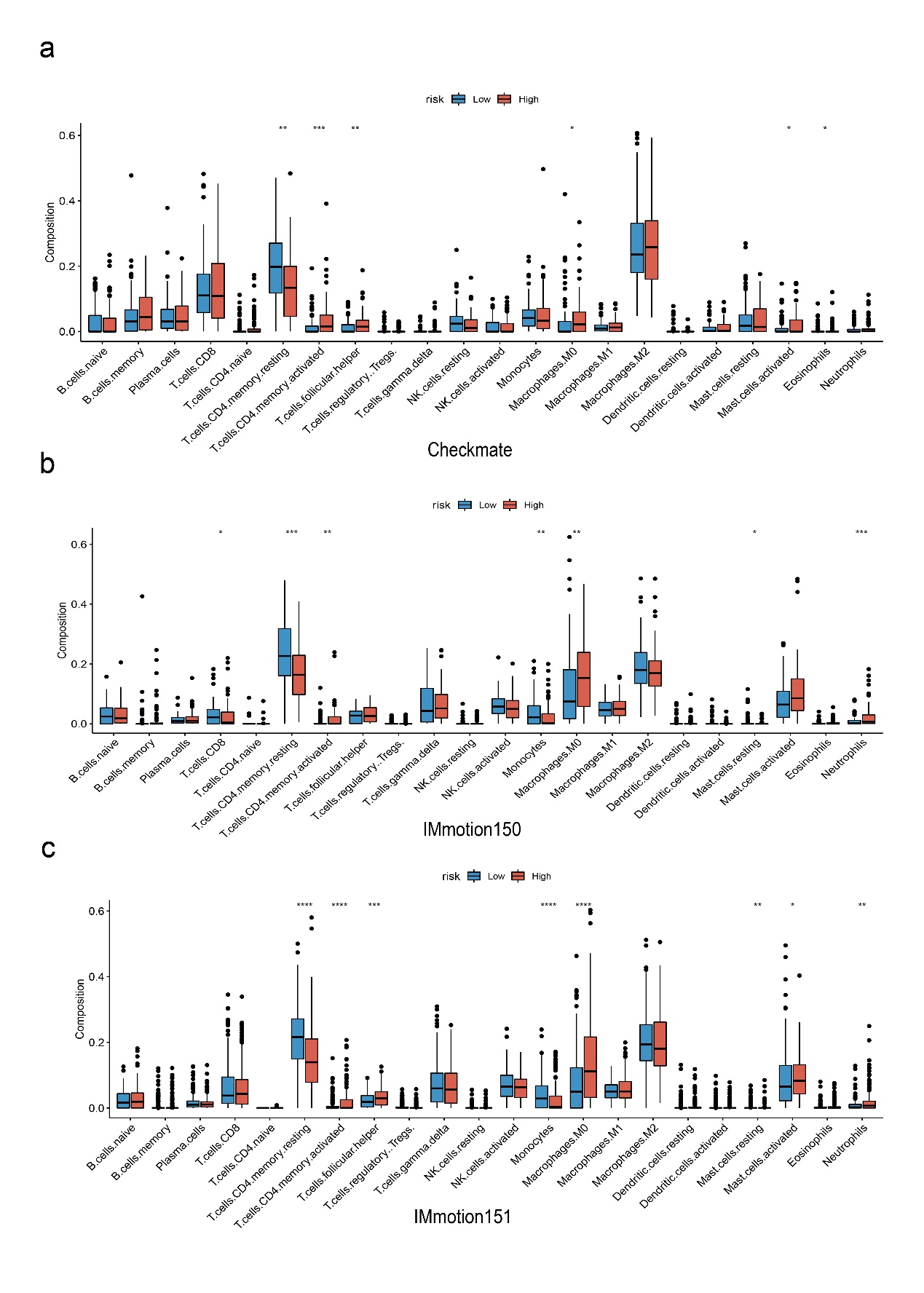


Figure S2. TME cell composition and fraction of individual immune cell types in three Immune-related cohorts. (a) the Nivolumab group of the CheckMate 025 study. (b) the Atezolizumab arm of IMmotion150. (c) the Atezolizumab plus Bevacizumab group of the IMmotion151 cohort.


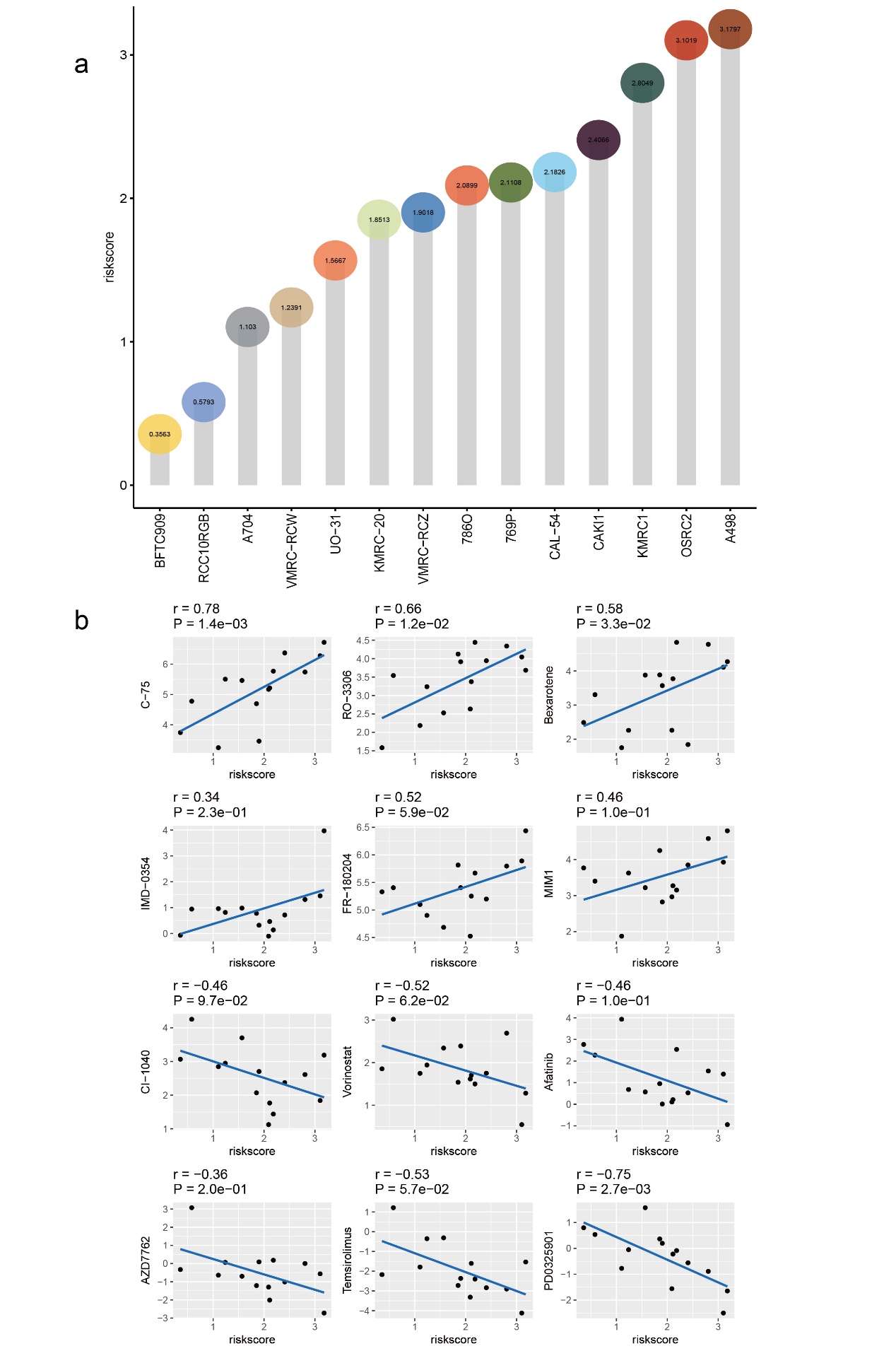


Figure S3. The risk score of ccRCC cell lines through CCLE and drug sensitivity data in GDSC. (a) A498 had the highest risk score while BFTC-909 had the lowest risk score. (b) The risk score had positive correlation with IC50 of C-75.


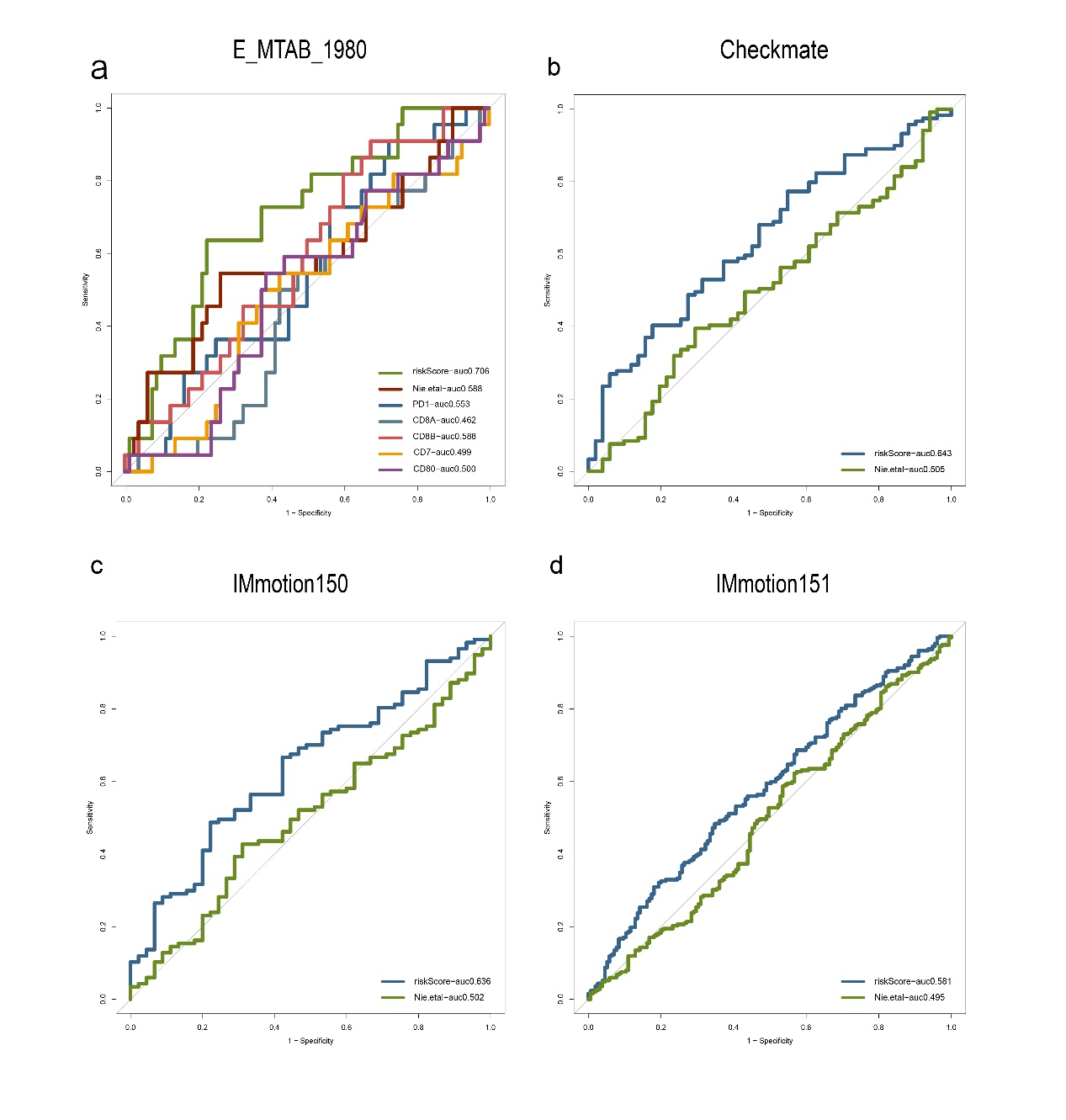


Figure S4. (a-d) The ROC curve of joint indicators and multi-model comparison in TCGA-KIRC cohort in E-MTAB-1980, the Nivolumab group of the CheckMate 025 study, the Atezolizumab arm of IMmotion150 and the Atezolizumab plus Bevacizumab group of IMmotion151 cohort.


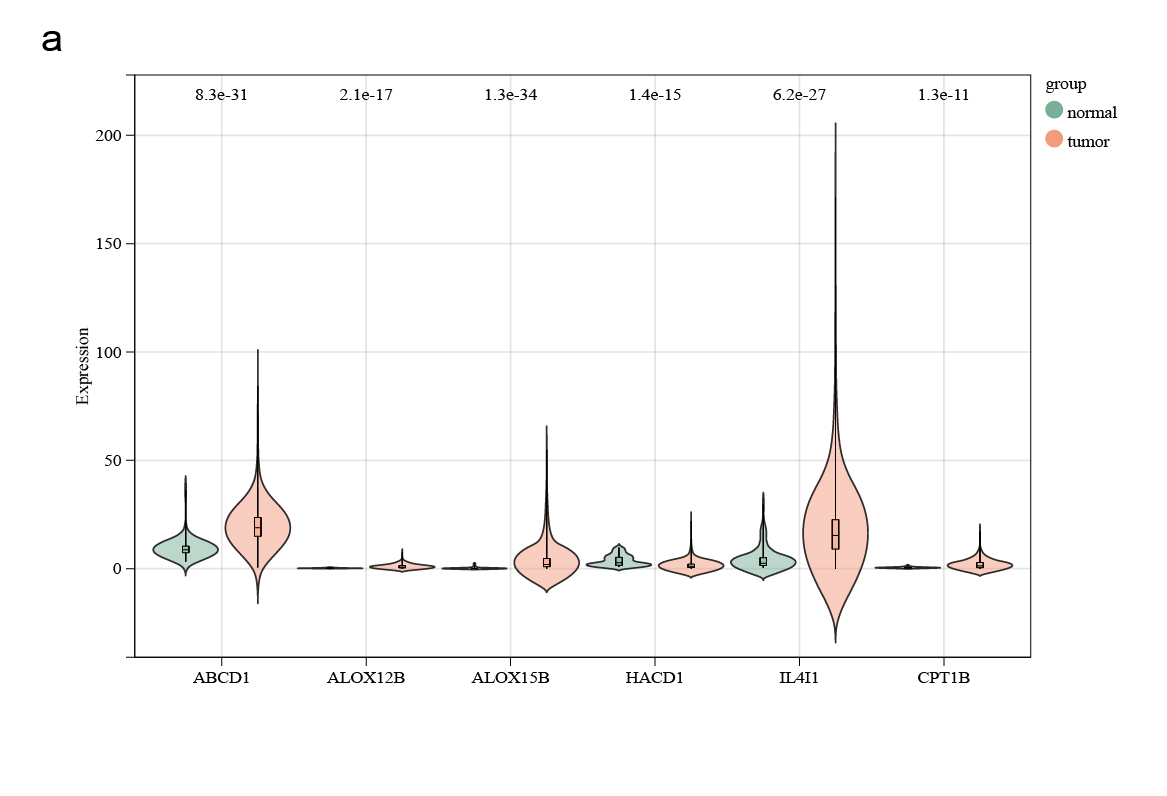
Figure S5. (a) mRNA expression of ABCD1, ALOX12B, ALOX15B, CPT1B, HACD1 and IL4I1 in TCGA-KIRC cohort.
