# Supplementary material for: Fatty acids metabolism affects the therapeutic effect of anti-PD-1/PD-L1 in tumor immune microenvironment in clear cell renal cell carcinoma
Source: J Transl Med. 2023 May 23;21:343. doi: 10.1186/s12967-023-04161-z (PMC10204332; doi:10.1186/s12967-023-04161-z)
Supplement: Supplementary file 2 — Additional file 2. Fatty acids metabolism-related genes. [file 12967_2023_4161_MOESM2_ESM.docx]

FADS2

RAP1GDS1

SDHA

ALDH2

ACSBG1

HSD17B4

HACD1

HSD17B3

PTGES2

DLD

CBR1

ADIPOR2

HPGD

CD1D

ERP29

PTGES

AADAT

PDHA1

ACAA2

ACSF3

MAOA

PRDX6

CD36

PRKAG2

ACADVL

ELOVL2

ALOX15B

PON1

MDH1

PECR

PPT2

GPX1

ENO3

ACBD4

CA6

CEL

CYP4F8

ACBD6

MORC2

SDHC

UBE2L6

CYP2C9

CYP4A22

ADH1A

DPEP3

ACBD5

AWAT1

ALOXE3

SLC25A1

EHHADH

ACACA

ALDH1A1

ACAA1

CA4

OLAH

PRKAA2

ACOT7

ACOT8

ABCD1

ACSL3

GLUL

ACOT9

NCAPH2

ADH5

HACD2

OSTC

CYP1B1

CYP4F22

FADS1

HMGCS2

IDH3G

ALOX15

MECR

CPOX

HSD17B11

ACSM6

HACD4

FAAH

CYP2U1

NTHL1

MIX23

SDHD

HSPH1

KMT5A

MID1IP1

ALDH1B1

PPARD

HTD2

SUCLA2

ADH4

UROD

PTGDS

HADHA

ABCC1

PTGR1

DHCR24

HCCS

FABP2

GPD1

PHYH

CYP2C8

ECI1

CYP2J2

RXRA

THEM4

FABP1

PDHB

PTGR2

ACOXL

NDUFAB1

CBR3

MCEE

PTGES3

CRYZ

PCTP

THEM5

CPT1B

LTA4H

CYP8B1

ADH1C

HSD17B8

BLVRA

SUCLG2

THRSP

ACOX3

ECH1

CYP1A2

GCDH

ELOVL3

PON3

TDO2

ACADS

SCD5

YWHAH

GGT5

TBXAS1

AMACR

PCCA

ACOT11

PTGS1

EPHX1

ACSBG2

ACSF2

ALDH3A1

ME1

FASN

DPEP1

GPX2

GAD2

AKR1C3

CYP4F3

MAPKAPK2

CBR4

MCAT

ODC1

ALAD

CIDEA

ELOVL4

CA2

RDH11

AOC3

GGT1

UROS

CYP2C19

LDHA

ACADM

ACOT13

HADHB

ADH6

ACADSB

ACSL1

ACAT1

G0S2

ELOVL1

CROT

GABARAPL1

ALOX5AP

NSDHL

FMO1

ACAT2

ALOX5

PTS

TECRL

GPX4

CPT1A

FAAH2

ECHS1

GSTZ1

ACSS1

HACD3

IDH3B

GRHPR

FH

ETFDH

ACSM3

PLA2G4A

ACAD10

HADH

ACBD7

SCP2

AUH

D2HGDH

SLC22A5

H2AZ1

SMS

XIST

ALOX12B

ELOVL5

NUDT7

ALDOA

VNN1

CYP4B1

MMUT

ACO2

UGDH

HSD17B12

MDH2

PPT1

DPEP2

EPHX2

ACOT6

PON2

PSME1

ACOT4

PTGS2

PTPRG

HAO2

ACOT2

SLC27A3

SERINC1

DLST

HSD17B10

ACLY

TECR

HMGCS1

ECI2

BPHL

METAP1

APEX1

ELOVL6

MIF

RETSAT

NUDT19

HPGDS

HSDL2

ADSL

PCCB

ACSL5

SCD

RDH16

PRXL2B

DECR2

SLC25A20

ACOX1

ACSL6

ACSL4

AQP7

IL4I1

ACADL

PTGIS

ALDH3A2

REEP6

HMGCL

ALDH7A1

ACAD11

INMT

ACACB

CPT1C

CYP4F11

ADH1B

ACOX2

DECR1

ENO2

CYP4F2

IDI1

ALDH9A1

HIBCH

LTC4S

ELOVL7

ALOX12

MMAA

NBN

LGALS1

GAPDHS

MGLL

SLC27A2

PPARA

HSP90AA1

MLYCD

CYP1A1

PCBD1

SLC25A17

BCKDHB

DBI

SUCLG1

CPT2

ACOT12

HACL1

ADH7

GPD2

TP53INP2

IDH1

CRAT

HSD17B7

CYP4A11

ACOT1

PRKAB2

BMPR1B
