# Supplementary material for: Fatty acids metabolism affects the therapeutic effect of anti-PD-1/PD-L1 in tumor immune microenvironment in clear cell renal cell carcinoma
Source: J Transl Med. 2023 May 23;21:343. doi: 10.1186/s12967-023-04161-z (PMC10204332; doi:10.1186/s12967-023-04161-z)
Supplement: Supplementary file 3 — Additional file 3. Three IL4I1 siRNAs sequence. [file 12967_2023_4161_MOESM3_ESM.docx]

stB0016927A genOFFTM st-h-IL4I1_001 GCATGACCTGGCAAAGGAA

stB0016927B genOFFTM st-h-IL4I1_002 CCAAGGTGTTCCTAAGCTT

stB0016927C genOFFTM st-h-IL4I1_003 GGCTCAACCTGACCAAGTT
