# Supplementary material for: Fatty acids metabolism affects the therapeutic effect of anti-PD-1/PD-L1 in tumor immune microenvironment in clear cell renal cell carcinoma
Source: J Transl Med. 2023 May 23;21:343. doi: 10.1186/s12967-023-04161-z (PMC10204332; doi:10.1186/s12967-023-04161-z)
Supplement: Supplementary file 4 — Additional file 4. All primers sequence. [file 12967_2023_4161_MOESM4_ESM.docx]

**Primer 5’ to 3’**

HACD1-1-F GCCTTGCCGCATGTGAAGAAAACAG

HACD1-1-R ACAATCACCTCTCCATGAAGCACCT

HACD1-2-F CCAGACATTTGCCTTGCTTGAGATCC

HACD1-2-R CCTGTTTTCTTCACATGCGGCAAGG

HACD1-3-F TCTTACAATATACGCTGCCTTGCCG

HACD1-3-R TCACCTCTCCATGAAGCACCTTTCT

ALXO15B-1-F TGCCCCACTGCCACCCTCTCTTCAA

ALXO15B-1-R AAGCGTTCCACTGCACCCCA

ALXO15B-2-F CCGCCGCTGTCACTACCTCCCAAA

ALXO15B-2-R AGGGTCATTGGGGCCGCAGAGAACT

ALXO15B-3-F ATGGTGGCCTCAGTGTTGGGTCCT

ALXO15B-3-R TCAGTGGGCAGGAAGATGGGGCTGT

ABCD1-1-F ATGGCCCGCATGTTCTACCACAGG

ABCD1-1-R AGCTTCTCGAACTTCCAGCCGCCCT

ABCD1-2-F ACTTCAAGAGGCCCAGGGAGCT

ABCD1-2-R TGCATGCCTTCCTCCACCCTGA

ABCD1-3-F TTGCCCGCAACCTCCTGACA

ABCD1-3-R AGCTCCCTGGGCCTCTTGAAGT

CPT1B-1-F TCCGCCAAACCCTGAAGCTGCT

CPT1B-1-R TGCCCACCATGACTTGAGCACCAGG

CPT1B-2-F TCAAGAAGTGCCGGACCAGCCCTGA

CPT1B-2-R AGGCTGTGGACTCGCTGGTACA

CPT1B-3-F ACCCAGAGCAGCACCCCAATCACCT

CPT1B-3-R GCTGGGCGTTCGTCTCTGAGCTTGA

ALOX12B-1-F AGAGACTTTGCAACTGGGGCGGTGG

ALOX12B-1-R TGTCTTTCCTGTGGCCTCCCGGAGT

ALOX12B-2-F GCTTTCAAAGTCCGCGGCCTGT

ALOX12B-2-R ACTTGTCTGGGATCCGCGTGCA

ALOX12B-3-F TGCACATTCCCAGTTACCGCCCTCC

ALOX12B-3-R TCCAACAGGCCGCGGACTTTGA

IL4I1-1-F AACCTGACCAAGTTCACCCA

IL4I1-1-R TCTGGTAGATGTCTTCGGGC

IL4I1-2-F GTGAAGCTGCGCAACTATGT

IL4I1-2-R TTCCCCTCCCCGAGAAGATA

IL4I1-3-F AGGCAGATAACAGGATCGGG

IL4I1-3-R GGCACCTTCTCCACCACATA
